# Neutrophil-to-Lymphocyte Ratio and Fibroblast Growth Factor 21: Their Role in Early Cardiovascular Involvement in Rheumatoid Arthritis

**DOI:** 10.3390/jcm14248844

**Published:** 2025-12-14

**Authors:** Mariusz Ciołkiewicz, Anna Kuryliszyn-Moskal, Ewa Jabłońska, Wioletta Ratajczak-Wrona, Mariusz Wojciuk, Piotr A. Klimiuk

**Affiliations:** 1 Department of Rehabilitation, Medical University of Bialystok, 24A M. Skłodowskiej-Curie St., 15-276 Bialystok, Poland; anna.kuryliszyn-moskal@umb.edu.pl (A.K.-M.); mariusz.wojciuk@umb.edu.pl (M.W.); 2 Department of Immunology, Medical University of Bialystok, 15A J. Waszyngtona St., 15-169 Bialystok, Poland; ewa.jablonska@umb.edu.pl (E.J.); wioletta.ratajczak-wrona@umb.edu.pl (W.R.-W.); 3 Department of Rheumatology and Internal Diseases, Medical University of Bialystok, 24A M. Skłodowskiej-Curie St., 15-276 Bialystok, Poland; piotr.klimiuk@umb.edu.pl

**Keywords:** NLR, FGF21, rheumatoid arthritis, LVDD, cardiac involvement

## Abstract

**Introduction:** Rheumatoid arthritis (RA) is associated with increased cardiovascular morbidity and mortality. Left ventricular diastolic dysfunction (LVDD) represents an early sign of cardiac involvement in RA. **Objectives:** This study aimed to evaluate the incidence of LVDD and the association of the neutrophil-to-lymphocyte ratio (NLR) and circulating FGF21 levels with chosen LVDD echocardiographic parameters, as well as to assess their diagnostic utility for LVDD in a cohort of patients with RA. **Patients and Methods:** A total of 51 RA patients (46 females, 5 males; average age 48.8 ± 8.2 years; median disease duration of 12 years) were enrolled. NLR and serum FGF21 levels were analysed for association with echocardiographic parameters of LVDD using univariate regression models. The diagnostic performance of these markers was evaluated by receiver operating characteristic (ROC) analysis. **Results:** LVDD was diagnosed in 10 patients (19.6%). The NLR was associated negatively with E velocity (β = −4.99, *p* = 0.02), E/A ratio (β = −0.16, *p* = 0.004), lateral and medial e′ velocities (β = −1.05, *p* = 0.038 and β = −0.97, *p* = 0.013, respectively), and positively with left atrial diameter (β = 2.08, *p* = 0.006). Serum FGF21 levels were negatively associated with the E/A ratio (β = −0.0005, *p* = 0.009) and lateral e′ velocity (β = −0.003, *p* = 0.04). ROC analysis demonstrated a greater diagnostic value for NLR (Youden index 0.30, cut-off point 2.26, sensitivity 50%, specificity 80%, and area under curve [AUC] 0.58) compared to FGF21 (Youden index 0.30, cut-off value 852.85 pg/mL, 100% specificity, 30% sensitivity, and AUC 0.48). **Conclusions:** NLR and FGF21 are associated with the echocardiographic parameters of the left ventricular diastolic dysfunction prior to the fulfilment of LVDD diagnostic criteria. RA patients with elevated NLR and FGF21 serum levels should be considered for LVDD screening.

## 1. Introduction

Rheumatoid arthritis (RA) is an autoimmune disease associated with a substantially increased risk of cardiovascular (CV) morbidity and mortality, estimated at approximately 50% higher than in the general population. To improve outcomes for RA patients, early detection of CV involvement, prior to the onset of myocardial infarction, stroke, or heart failure, is essential. Timely diagnosis of cardiac involvement in RA enables prompt therapeutic intervention and risk factor modification, potentially preventing major adverse cardiovascular events and their complications.

Several factors may contribute to myocardial dysfunction and cardiac damage in patients with rheumatoid arthritis. The first potential mechanism is premature atherosclerosis leading to coronary artery disease and, consequently, myocardial ischemia. Formation of atherosclerotic plaques in coronary arteries in RA may result from the combined influence of traditional non-modifiable (e.g., age, sex, family history, ethnicity) and modifiable (e.g., hyperlipidaemia, arterial hypertension, diabetes mellitus, cigarette smoking) risk factors, as well as disease-specific factors (e.g., chronic systemic inflammation, oxidative stress, endothelial dysfunction, and the use of glucocorticoids or non-steroidal anti-inflammatory drugs).

In rheumatoid arthritis, activation and death of neutrophils is associated with the formation of neutrophil extracellular traps (NETs), web-like structures composed of histones, DNA, and antimicrobial proteins, inter alia. This process may be stimulated by cytokines and immune complexes and is dependent on reactive oxygen species. While NETs may trap and neutralise proinflammatory cytokines, they also exert cytotoxic and prothrombotic effects, linking inflammation and coagulation. Additional pathogenic effects of NETs include direct endothelial cell dysfunction and damage, as well as the embedding of NETs into atherosclerotic plaques, promoting plaque instability and thrombosis.

In the pathogenesis of RA, several proinflammatory cytokines, especially tumour necrosis factor α, Interleukin-1, and Interleukin-6, as well as reactive oxygen species, may lead to endothelial dysfunction and vascular injury. Imbalance between antioxidant mechanisms and reactive oxygen species results in oxidative stress, leukocyte migration and differentiation, oxidative modification of lipoproteins, and promotion of atheromatic plaque formation and progression.

Endothelial dysfunction with expression of proinflammatory cytokines and adhesion molecules may, in turn, amplify proinflammatory and prothrombotic states, as well as atherosclerotic plaque formation. Immune mechanisms, endothelial dysfunction, and vascular injury may lead to microcirculation dysfunction on the one hand and premature atherosclerosis on the other, even in RA patients without traditional cardiovascular risk factors.

Other mechanisms of myocardial dysfunction are not the direct result of ischemia but are related to the direct impact of chronic inflammation. Chronic inflammation-related myocardial remodelling is caused by deposition of collagen and other extracellular matrix components, leading to increased stiffness, cardiomyocyte hypertrophy, and, consequently, diminished relaxation and diastolic dysfunction. Proinflammatory cytokines such as tumour necrosis factor α, Interleukin-1, and Interleukin-6 play a major role in this process by activating cardiac fibroblasts, promoting extracellular matrix deposition, impairing calcium handling and contractile function, and altering extracellular matrix turnover (imbalance between metalloproteinases and their inhibitors).

Dysfunction of coronary microcirculation, driven by CV risk factors and chronic inflammation, may impair coronary flow reserve and lead to ischemia with subsequent cardiomyocyte autophagy, apoptosis, necrosis, and collagen deposition in the interstitial space. This process may lead to the development of diastolic dysfunction even in individuals without epicardial stenosis. In severe cases, myocardial dysfunction may progress from diastolic to systolic dysfunction [1,2,3,4,5].

Left ventricular diastolic dysfunction (LVDD) is recognised as an early manifestation of cardiac involvement, predating clinical signs and symptoms of heart failure (HF). Diastolic dysfunction occurs more frequently than systolic dysfunction in RA and is more prevalent than in healthy individuals [6,7]. The reported prevalence of LVDD in RA patients ranges from 13% to 47% [8,9,10,11,12,13,14]. Longitudinal studies have demonstrated a 24% one-year progression rate from normal diastolic function to mild dysfunction in RA cohorts, and an increase in LVDD prevalence from 40.7% to 57.9% during 4–6 years of follow-up [15,16].

Surrogate biomarkers may improve early detection in asymptomatic patients. The neutrophil-to-lymphocyte ratio (NLR) is a readily accessible and cost-effective indicator of systemic inflammation, calculated by dividing neutrophil count by the lymphocyte count from complete blood counts. NLR reflects the balance between the innate immune response (neutrophils) and the adaptive immune response (lymphocytes) during several pathological conditions. In adults, a normal NLR range is between 1 and 2; values between 2.3 and 3.0 are considered a grey zone, while values below 0.7 or above 3.0 are regarded as abnormal [17].

Several studies have demonstrated a positive association between NLR and diastolic dysfunction in patients with type 2 diabetes mellitus [18], hyperthyroidism [19], heart failure with preserved ejection fraction (HFpEF) [20], and pulmonary hypertension [21]. Neutrophil degranulation is linked to diastolic dysfunction in diabetes [22], and neutrophiles are pivotal in adverse cardiac remodelling in experimental heart failure models [23]. While the role of NLR in RA diagnosis, and its association with clinical characteristics, particularly disease activity [24,25,26,27,28,29,30], and response to treatment [31,32,33] has been studied, its utility in predicting diastolic dysfunction in RA remains unexplored.

Fibroblast Growth Factor 21 (FGF21) belongs to the family of organokines, the group of signalling molecules, regulating metabolism, mediating inter-organ communication, and acting via endocrine, autocrine, or paracrine pathways [34]. Organokines are classified by their tissue of origin, including adipokines (adipose tissue), myokines (muscle), hepatokines (liver), and osteokines (bone). In RA, organokines contribute to immune dysregulation, systemic inflammation, and pathological angiogenesis, enhancing cytokines and metalloproteinases production, which promotes cartilage degradation and radiographic damage [34]. FGF21 is synthesised in adipose tissue, liver, and muscle in response to stimuli such as physical exercise, emotional stress, nutrient excess or deficiency, or cold exposure. Physiologically, FGF21 regulates glucose and lipid metabolism, enhancing insulin sensitivity and glucose uptake, controlling lipolysis, and attenuating lipogenesis [35,36]. Recently, FGF21 has been shown to exert protective effects on the myocardium. Increases in serum FGF21 are observed in coronary artery disease, cardiac hypertrophy, and diabetic cardiomyopathy, often paralleling rises in brain natriuretic peptide (BNP) as part of a compensatory response. Experimental FGF21 administration mitigates progression of forementioned diseases. [37]. FGF21 attenuates inflammation and oxidative stress, inhibits cardiac hypertrophy and collagen synthesis, and may counteract myocardial remodelling, cellular senescence, apoptosis, pyroptosis, and ferroptosis of cardiomyocytes [35].

Recent studies [38] have linked FGF21 levels to left ventricular diastolic dysfunction, HFpEF, and have reported predictive utility in dilated cardiomyopathy [39] and heart failure with reduced ejection fraction (HFrEF) [40].

Although elevated circulating FGF21 levels have been observed in RA and associated with altered body composition, reduced physical function, and increased inflammatory cytokines [41,42,43], its prognostic value for LVDD and heart failure in RA has not been investigated.

The aim of this study was to investigate the incidence of LVDD and the association of the NLR and circulating FGF21 levels with chosen LVDD echocardiographic parameters, as well as to assess their diagnostic utility for LVDD in a cohort of patients with RA.

## 2. Population Characteristics

Fifty-one RA patients, fulfilling the 2010 ACR/EULAR classification criteria [44], were enrolled in this study. The study protocol received approval from the Bioethics Committee of the Medical University of Bialystok, Poland (approval number R-I-002/234/2016; 30 June 2016). Participants were recruited between April 2017 and August 2019 at the Department of Rehabilitation of the Medical University of Bialystok, Poland. Written informed consent was obtained from all participants prior to study enrolment. All procedures were conducted in accordance with the Declaration of Helsinki.

Demographic and anthropometric data including sex, age, height, and weight were collected. Medical history included RA duration, morning stiffness, comorbidities, treatment patterns, and smoking status. Radiographs of hands and feet were assessed, and disease activity was evaluated using the Clinical Disease Activity Index (CDAI) [45].

Laboratory assessments comprised complete blood count, estimated glomerular filtration rate (eGFR, MDRD equation), rheumatoid factor, and high sensitivity C-reactive protein (hsCRP) (Roche Diagnostics, Rotkreuz, Switzerland). Serum concentrations of Fibroblast Growth Factor 21 and Brain Natriuretic Peptide were determined from samples stored at −80 °C using commercially available ELISA kits (R&D Systems, Minneapolis, MN, USA and Elabscience, Wuhan, China, respectively), according to the manufacturers’ protocols.

## 3. Echocardiographic Assessment of Diastolic Dysfunction

Comprehensive transthoracic echocardiography (TTE) was performed using ClearVue 550 ultrasound system (Philips Healthcare, Best, Netherlands) by a single experienced cardiologist, following recommendations of the American Society of Echocardiography/European Association of Cardiovascular Imaging [46,47]. Assessment of left ventricular diastolic dysfunction was based on two-dimensional and Doppler echocardiography. Left atrial anteroposterior diameter (LAD) was measured in the parasternal long-axis view perpendicular to the aortic root at the level of the aortic sinuses. Left atrial volume (LAV) was calculated using the biplane disc summation (modified Simpson’s) method in both the apical 4-chamber and 2-chamber views at end-systole. Left atrial volume index (LAVI) was computed as LAV indexed to body surface area (BSA). Mitral inflow peak E- and A-wave velocities were measured with pulsed-wave Doppler in the apical 4-chamber view at the mitral leaflet tips; E/A ratio was calculated as the ratio of these velocities. Lateral and septal mitral annular velocities were acquired in the apical 4-chamber view with tissue Doppler imaging; the pulsed-wave sample volume was placed at the lateral and septal insertion points of the mitral annulus, adjacent to the ventricular myocardium, to measure maximal early diastolic (e′) velocities. The E/e′ ratio was calculated by dividing mitral peak E-wave velocity by the mean value of the medial and lateral e′ velocities. Tricuspid regurgitation velocity was assessed in the apical four-chamber view. First, colour Doppler was used to identify the tricuspid regurgitation jet. Subsequently, a continuous-wave Doppler cursor was aligned along the path of the regurgitant jet to obtain the highest velocity signal.

Left ventricular diastolic dysfunction was classified according to current European Society of Cardiology guidelines [47]. Grade I diastolic dysfunction was diagnosed in patients with E/A ≤ 0.8 and E < 50 cm/s, or E/A ≤ 0.8 with E > 50 cm/s, when none or only one of the three following criteria was positive: average E/e′ > 14, tricuspid regurgitation velocity > 2.8 m/s, or LAVI > 34 mL/m^2^. Grade II dysfunction was diagnosed in subjects with E/A ≤ 0.8 and E > 50 cm/s, or E/A > 0.8 and <2, with two or all positive of the aforementioned three criteria, and grade III for E/A ≥ 2.

## 4. Statistical Analysis

All statistical analyses were performed using R (version 4.4.2) and STATISTICA (version 13-3), with a significance threshold of α = 0.05. Depending on distribution, continuous variables were presented as mean ± SD or median (IQR). Normality of distribution was assessed with the Shapiro–Wilk test, skewness, and kurtosis. Levene’s test was applied to assess homogeneity of variance.

To examine the impact of predictors on left ventricular diastolic dysfunction parameters, linear regression approach was applied. Spearman correlation was used to assess the correlation between measured markers and between brain natriuretic peptide and parameters of diastolic dysfunction. To evaluate the diagnostic values of the markers with respect to LVDD, a receiver operator characteristic curve was used with counting Youden index, cut-off values, sensitivity, specificity, and area under curve (AUC).

## 5. Results

A total of 51 participants (46 female and 5 male), aged 48.80 ± 8.20 years with a median disease duration of 12 years (interquartile range, 4.95; 20.25), were enrolled in the study. Approximately half of individuals (50.9%) presented with erosive disease, and 40 (78.4%) were seropositive. None of the patients had a diagnosis of heart failure previous to the entry into the research study; twelve (23.5%) were diagnosed with arterial hypertension, of whom only one was not receiving antihypertensive therapy. Only one patient had type 2 diabetes. Conventional synthetic disease-modifying antirheumatic drugs (csDMARDs) were administered to 98% of patients, with the majority receiving methotrexate. Biological DMARDs (bDMARDs) were used in 32 patients (62.7%), and corticosteroids in 11 patients (21.6%).

The median hsCRP level was 2.88 mg/L (1.06–6.19), indicating low laboratory disease activity; similarly, the median value of NLR was relatively low at 1.78 (1.13–2.26). In contrast, the mean CDAI value was 25.00 ± 12.12, consistent with moderate clinical disease activity. Mean haemoglobin (13.15 ± 1.25 g/dL) and eGFR (92.08 ± 14.85 mL/min/1.73 m^2^) were within normal ranges. The median FGF 21 level was 98.13 pg/mL (42.96–187.72), while the median BNP level was relatively high at 64.37 pg/mL (46.87–97.21). Detailed baseline characteristics of the study population, including selected echocardiographic parameters of left ventricular diastolic dysfunction, are presented in Table 1.

## 6. Diastolic Dysfunction and Selected Echocardiographic Parameters

Left ventricular diastolic dysfunction was identified in ten patients (19.6%), and in all cases, it was mild (grade I LVDD). Among the patients with LVDD, five (50%) had hypertension and seven (70%) were smokers. Only one enrolled diabetic patient did not have LVDD.

The mean E/A ratio was within the normal range (1.20 ± 0.35), as were peak E-wave and peak A-wave velocities (65.5 ± 13.2 and 57.49 ± 13.87, respectively). Both mean lateral (13.25 ± 3.14) and septal (11.29 ± 2.45) mitral annular velocities were normal, as well as the median E/e′ ratio (5.20; 4.60–6.05). The left atrium was not enlarged, as measured in parasternal long-axis view (35.16 ± 4.77 mm), left atrial volume (43.1 ± 14.6 mL), and left atrial volume index (43.1 ± 14.6 mL/m^2^).

## 7. BNP, NLR, FGF21, and Diastolic Dysfunction

There was no statistically significant correlation between BNP levels and either NLR or FGF21 (*p* = 0.221 and *p* = 0.786, respectively). Moreover, BNP concentration was not correlated with the presence of LVDD (*p* = 0.481). None of the echocardiographic parameters correlated with BNP serum concentrations: E-wave velocity (*p* = 0.962), E/A ratio (*p* = 0.761), lateral e′ velocity (*p* = 0.767), septal e′ velocity (*p* = 0.884), E/e′ ratio (*p* = 0.932), LAD (*p* = 0.428), and LAVI (*p* = 0.815).

## 8. Association of NLR and FGF21 with Echocardiographic Parameters of LVDD

Neutrophil-to-lymphocyte ratio was negatively associated with E-wave velocity (β = −4.83, *p* = 0.019). Each increase in NLR by one unit decreased E/A ratio by 0.15 (*p* = 0.005). NLR was negatively associated with both lateral and septal mitral annular velocities (β = −1.01, *p* = 0.039, and β = −0.94, *p* = 0.014, respectively). No association with E/e′ ratio (*p* = 0.945) was found. Regarding parameters of left atrial enlargement, a positive association was found only between NLR and left atrial long-axis diameter (LAD); each unit increase in NLR raised LAD by 2.01 mm.

Fibroblast growth factor 21 was negatively associated with E/A ratio; each unit increase in FGF21 reduced E/A by 0.0005 (*p* = 0.009). There was a trend toward negative association between FGF21 and E-wave velocity of left ventricular filling (β = −0.01, *p* = 0.053). FGF21 was negatively associated with lateral mitral annular velocity (β = −0.003, *p* = 0.042), with a trend noted for septal mitral annular velocity (β = −0.002, *p* = 0.125). No association was observed with E/e′ ratio (*p* = 0.515). There was only a trend toward positive association with LAD (β = −0.0004, *p* = 0.107).

**Figure 1:**
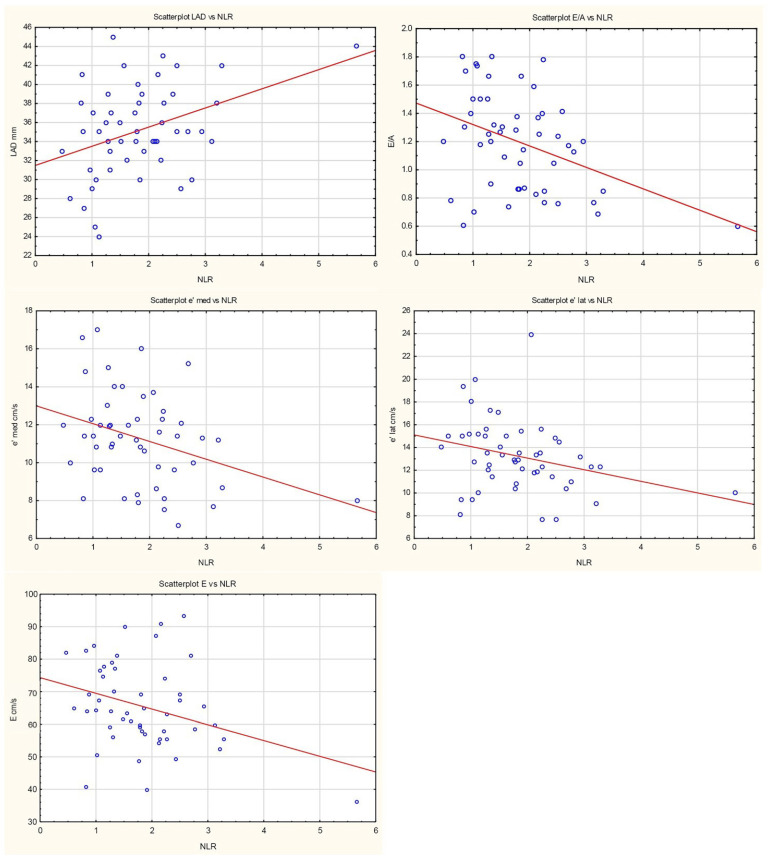
Scatterplots presenting the relationships between NLR and LVDD. The regression line marked in red.

**Figure 2:**
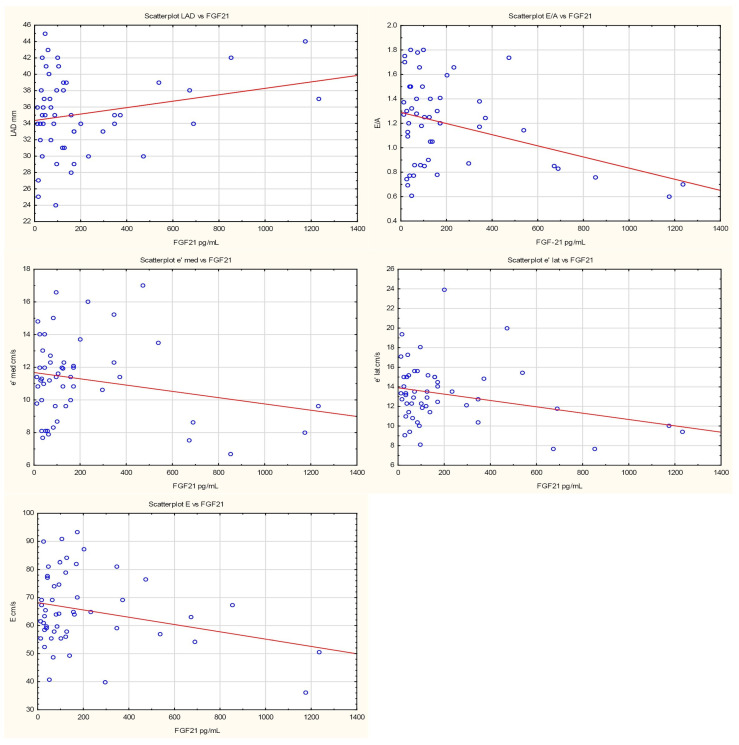
Scatterplots presenting the relationships between FGF21 levels and LVDD. The regression line marked in red.

Figure 1 and Figure 2 and Table 2 and Table 3 summarise the relationships between NLR, FGF21 concentrations, and echocardiographic parameters of left ventricular diastolic dysfunction.

## 9. Receiver Operating Characteristic (ROC) Curves

Receiver operating characteristic (ROC) analysis of the diagnostic value of the neutrophil-to-lymphocyte ratio for detecting left ventricular diastolic dysfunction in patients with rheumatoid arthritis yielded at the cut-off value of 2.26, with a Youden index of 0.30. The sensitivity and specificity were 50% and 80%, respectively. The area under the curve (AUC) was 0.58.

The diagnostic value of FGF21 for LVDD in RA patients was lower, with a Youden index of 0.30, a cut-off value of 852.85 pg/mL, high specificity (100%), low sensitivity (30%), and a low AUC of 0.48.

**Figure 3:**
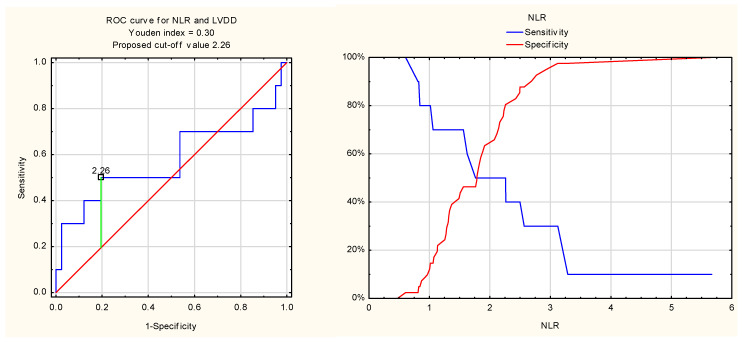
ROC curve for NLR and LVDD.

**Figure 4:**
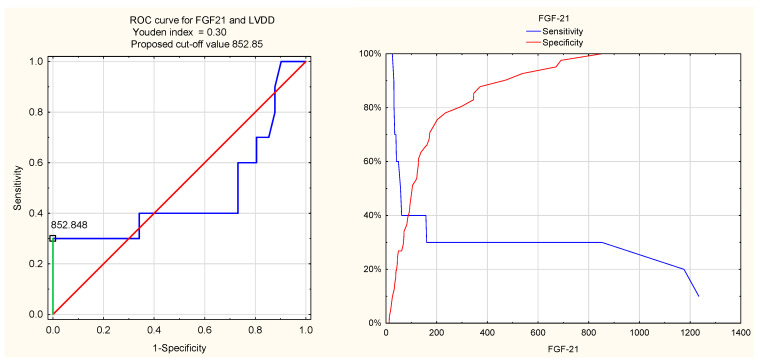
ROC curve for FGF21 and LVDD.

The ROC curves for NLR and FGF21 are shown in Figure 3 and Figure 4.

For BNP, the Youden index value was low (0.13), with a cut-off level of 92.21 pg/mL, sensitivity 40%, specificity 73%, and an AUC of 0.57. These values suggest low diagnostic utility of BNP for LVDD in the rheumatoid arthritis population.

## 10. Discussion

In the present study, NLR values were significantly negatively associated with mitral inflow E velocity (*p* = 0.019), E/A ratio (*p* = 0.005), and both lateral and septal e′ velocities (*p* = 0.039 and *p* = 0.014, respectively); in contrast, NLR showed a positive association with No long-axis left atrial diameter (*p* = 0.006). Serum FGF21 concentrations were negatively associated with the E/A ratio (*p* = 0.009) and lateral e′ velocity (*p* = 0.042), demonstrated only a trend toward negative association with E velocity (*p* = 0.053) and septal e′ velocity (*p* = 0.125), as well as positive association with LAD (*p* = 0.107). These findings may be attributable to the relatively small sample size (n = 51).

Although NLR was associated with several echocardiographic parameters of LVDD, its diagnostic performance in identifying LVDD was relatively limited. Similarly, despite significant associations between FGF21 and the E/A ratio as well as lateral e′ annular velocity, FGF21 did not demonstrate diagnostic utility for LVDD in ROC analysis. This may be explained by the hypothesis that NLR and FGF21 are sensitive markers reflecting early unfavourable changes in diastolic function, preceding the fulfilment of LVDD diagnostic criteria.

While previous studies have investigated the prevalence of LVDD among RA patients and the link between NLR, FGF21 serum levels, RA clinical characteristics, and treatment outcomes, no prior research has specifically addressed the diagnostic values of NLR and FGF21 for LVDD assessment in RA. To the best of our knowledge, this is the first study to evaluate the relationship between NLR, circulating FGF21 concentrations, and echocardiographic parameters of diastolic dysfunction and LVDD prevalence in the RA population.

The incidence of left ventricular diastolic dysfunction in our cohort was 19.6%. This rate exceeds the 13% reported by Rodrigues et al. [8], where the RA cohort was older (median age, 58 years; females, 78%) compared to the present study population (mean age, 48.8 years; females, 90.2%). The prevalence of arterial hypertension was 43% in the overall RA population and 50% among LVDD patients in Rodrigues et al., whereas in our cohort, hypertension prevalence was lower (23.5%) and identical in the LVDD subgroup.

Another study [11] identified a higher LVDD incidence of 31% in the RA group with low disease activity; these patients were slightly older (mean age, 53.9 years, female prevalence, 77%). Notably, hypertensive subjects were excluded (in our study, 23.5% had hypertension). Partial explanation of this discrepancy (no hypertensive patients, but higher LVDD incidence) may be due to their reliance on the E/A ratio alone for LVDD diagnosis and a cut-off value of <1.0 instead of 0.8, as recommended by current guidelines.

In a study of HFpEF patients [20], positive associations were reported between NLR, NT-proBNP, and mitral E/e′ ratio, with good prediction of HFpEF (ROC AUC, 0.796). In our RA cohort, NLR was negatively associated with E velocity, E/A ratio, and both e′ lateral and septal velocities, and positively with left atrial diameter, but showed no link to E/e′ ratio. No cases of heart failure were diagnosed, and the predictive value of NLR for LVDD was relatively small (AUC 0.58).

Li et al. [30] recommended an NLR cut-off > 2.258 as a risk factor for moderate-to-severe pain and higher disease activity in RA patients. In our study, a similar value (2.26) was identified by ROC analysis for LVDD diagnosis. Further research is required to clarify the relationship between NLR, RA clinical features, and LVDD prevalence.

Chou et al. [48] observed that in HFpEF patients, both FGF21 and NT-pro-BNP were positively correlated with left atrial dimension and the E/e′ ratio. Moreover, serum FGF21 was significantly associated with NT-pro-BNP, and regression analyses demonstrated that log FGF21 and log NT-pro-BNP were linked to the E/e′ ratio. In our study of RA patients, FGF21 was negatively associated with the E/A ratio and lateral e′ velocity, showed only a trend with E velocity and septal e′ velocity, and was positively associated with LAD. No correlations were found between FGF21 and BNP, nor between BNP and any of LVDD echocardiographic parameters. This finding may reflect the absence of heart failure diagnoses in our sample.

In our study, BNP levels were relatively high (median, 64.37 pg/mL; IQR, 46.87–97.21), yet patients exhibited no signs or symptoms of heart failure. BNP concentrations were not correlated with LVDD or any LVDD echocardiographic indices, consistent with prior studies suggesting that increased BNP or NT-proBNP levels in RA are independent of traditional cardiovascular risk factors or left ventricular abnormalities [49,50].

The present study has several limitations. Firstly, the relatively small number of participants (n = 51) may limit the statistical power and generalizability. Secondly, there was no healthy control group for comparison. Thirdly, only traditional 2D, pulsed Doppler, and tissue Doppler techniques were used; advanced methods such as global longitudinal strain, which sensitively detect early systolic dysfunction in asymptomatic patients, were not employed.

Further research is necessary to more accurately determine the diagnostic and prognostic value of NLR and FGF21 in relation to left ventricular diastolic dysfunction and heart failure among patients with rheumatoid arthritis.

## 11. Conclusions

NLR and FGF21 are associated with echocardiographic parameters of left ventricular diastolic dysfunction prior to the fulfilment of LVDD diagnostic criteria. RA patients with elevated NLR and FGF21 levels should be considered for LVDD screening. BNP does not demonstrate predictive value for either echocardiographic parameters of left ventricular diastolic dysfunction or LVDD presence.

## Figures and Tables

**Table 1:** Study participant characteristics.

Variable	Mean ± SD/Median (IQR)/*n* (%)
Sex, female	46 (90.2)
Age, years	48.80 ± 8.20
Disease duration, years	12 (4.95–20.25)
Morning stiffness, min	30 (10–60)
Current smoking	12 (23.5)
Erosive disease	26 (50.9)
CDAI	25.00 ± 12.12
RF	40 (78.4)
Medication:	
Methotrexate	46 (90.2)
Corticosteroids	11 (21.6)
Biologics	32 (62.7)
Arechine	1 (1.9)
Cyclosporine	1 (1.9)
Sulphasalazine	2 (3.9)
Leflunomide	1 (1.9)
NSAIDS	28 (54.9)
Statins	3 (5.7)
Antihypertensive drugs	11 (21.6)
Body weight, kg	71.24 ± 15.66
Height, cm	164.00 (160.00–168.00)
BMI, kg/m^2^	26.30 ± 5.25
Comorbidities:	
Hypertension	12 (23.5)
Thyroid nodules	2 (3.9)
Asthma	2 (3.9)
Hashimoto disease	3 (5.9)
Depression	5 (9.8)
Hyperlipidaemia	2 (3.9)
Gout	1 (1.9)
Type 2 diabetes	1 (1.9)
Antiphospholipid syndrome	1 (1.9)
Chronic kidney disease	1 (1.9)
Goitre	1 (1.9)
NLR	1.78 (1.13–2.26)
FGF21, pg/mL	98.13 (42.96–187.72)
hsCRP mg/L	2.88 (1.06–6.19)
BNP pg/mL	64.37 (46.87–97.21)
Haemoglobin g/dL	13.15 ± 1.25
eGFR mL/min/1.73 m^2^	92.08 ± 14.85
E/A	1.20 ± 0.35
E cm/s	65.5 ± 13.2
A cm/s	57.49 ± 13.87
e′ lat cm/s	13.25 ± 3.14
e′ med cm/s	11.29 ± 2.45
E/e′	5.20 (4.60–6.05)
LAD mm	35.16 ± 4.77
LAV mL	43.1 ± 14.6
LAVI mL/m^2^	23.82 ± 6.83

Data are presented as mean and (SD) standard deviation or median and (IQR) interquartile range. Abbreviations: A, peak A-wave velocity; BMI, body mass index; BNP, brain natriuretic peptide; CDAI, Clinical Disease Activity Index; E, peak E-wave velocity; e′ lat, lateral mitral annular velocity; e′ med, septal mitral annular velocity; eGFR, estimated glomerular filtration rate; FGF21, fibroblast growth factor 21; hsCRP, high sensitivity C-reactive protein; LAD, left atrium long-axis diameter; LAV, left atrial volume; LAVI, left atrial volume index; NLR, neutrophil-to-lymphocyte ratio; NSAIDS, non-steroidal anti-inflammatory drugs; RF, rheumatoid factor.

**Table 2:** Associations of NLR values with echocardiographic parameters of diastolic dysfunction.

	Univariate Analysis		
	β (95% CI)	Std β	*p*
E, cm/s	−4.83 (−8.85, −0.82)	−0.33	0.019
A, cm/s	2.77 (−0.37, 4.74)	0.18	0.210
E/A	−0.15 (−0.25, −0.05)	−0.39	0.005
e′ lat, cm/s	−1.01 (−1.99, −0.05)	−0.29	0.039
e′ med, cm/s	−0.94 (−1.68, −0.20)	−0.34	0.014
E/e′	−0.01 (−0.42, 0.39)	−0.001	0.945
LAD, mm	2.01 (0.59, 3.43)	0.38	0.006
LAV, mL	3.12 (−1.48, 7.73)	0.19	0.179
LAVI, mL/m^2^	0.96 (−1.22, 3.14)	0.12	0.382

Abbreviations: A, peak A-wave velocity; CI, confidence interval; E, peak E-wave velocity; e′ lat, lateral mitral annular velocity; e′ med, septal mitral annular velocity; LAD, left atrium long-axis diameter; LAV, left atrial volume; LAVI, left atrial volume index; std. β, standardised beta.

**Table 3:** Associations of FGF21 serum levels with echocardiographic parameters of diastolic dysfunction.

	Univariate Analysis		
	β (95% CI)	Std β	*p*
E, cm/s	−0.01 (−0.026; 0.00015)	−0.27	0.053
A, cm/s	0.01 (0.002; 0.023)	0.24	0.095
E/A	−0.0005 (−0.0008; −0.0001)	−0.36	0.009
e′ lat, cm/s	−0.003 (−0.006; −0.0001)	−0.29	0.042
e′ med, cm/s	−0.002 (−0.004; 0.0006)	−0.22	0125
E/e′	0.004 (−0.0009; 0.002)	0.09	0.515
LAD, mm	0.004 (−0.0009; 0.009)	0.23	0.107
LAV, mL	0.006 (−0.009; 0.021)	0.18	0.410
LAVI, mL/m^3^	0.003 (−0.004; 0.099)	0.12	0.420

Abbreviations: A, peak A-wave velocity; CI, confidence interval; E, peak E-wave velocity; e′ lat, lateral mitral annular velocity; e′ med, septal mitral annular velocity, LAD, left atrium long-axis diameter; LAV, left atrial volume; LAVI, left atrial volume index; std. β, standardised beta.

## Data Availability

Data available on request from authors.
